# Dysbiosis associated with enhanced microbial mobility across the respiratory tract in pulmonary tuberculosis patients

**DOI:** 10.1186/s12866-025-04206-x

**Published:** 2025-08-12

**Authors:** Mingyang Qin, Weimin Ding, Lin Qin, Ruobing Liang, Yang Guo, Ying Zhao, Huifang Xu, Yanhua Wen, Yu Pang, Liang Li

**Affiliations:** 1 Department of Epidemiology, School of Public Health, Cheeloo College of Medicine, Shandong University, Jinan, 250012 P.R. China; 2 Department of Bacteriology and Immunology, Beijing Chest Hospital, Capital Medical University/Beijing Tuberculosis & Thoracic Tumor Research Institute, Beijing, 101149 P.R. China; 3 Department of Endoscopic Diagnosis & Treatment, Beijing Chest Hospital, Capital Medical University/Beijing Tuberculosis & Thoracic Tumor Research Institute, Beijing, 101149 P.R. China; 4 Department of Research and Development, Hugobiotech Co., Ltd., Beijing, 100176 China

**Keywords:** Pulmonary tuberculosis, Respiratory tract, Microbiota, Interaction

## Abstract

**Background:**

The microbiota is actively engaged in interaction networks both with the host and among its own constituent members. However, comprehensive studies examining the microbiome profiles across various respiratory sites in pulmonary tuberculosis (PTB) are lacking. Here, we explored the diversity of the microbiome in PTB patients across multiple respiratory sites and investigated potential interactions between the microbiomes of these sites.

**Methods:**

A total of 130 respiratory tract samples were collected from multiple sites of 22 patients with PTB and 14 healthy individuals, including the oral cavity, trachea, and both the healthy and affected sides of the lungs. These samples were subjected to metagenomic sequencing to analyze the characteristics and diversity of the respiratory microbiome.

**Results:**

We found that the respiratory tract of PTB patients had higher microbial diversity than seen in the healthy individuals (8,182 vs 6,465). Among them, *Rothia*, *Prevotella* and *Actinomyces* exhibited higher proportions in PTB. The characteristics of high diversity features in the oral site were more prominent with PTB, especially the notable difference of *Rothia mucilaginosa*. Additionally, *Streptococcus*, *Neisseria*, *Prevotella* and *Fusobacterium* have strong interactions with other species at present at various sites of PTB patients, as well as frequent communication between these species during migration in the upper and lower respiratory tract.

**Conclusions:**

The diversity and translocation of microbiota across the respiratory tract in PTB patients are associated with increased susceptibility of microbiome. The predominance of *Rothia*, *Prevotella*, and *Actinomyces* may represent progression-associated microbial signatures, warranting mechanistic studies on their pathogenic potential through host-microbe interactions to guide therapeutic targeting.

**Supplementary Information:**

The online version contains supplementary material available at 10.1186/s12866-025-04206-x.

## Background

Tuberculosis (TB), a severe chronic and serious infectious disease caused by *Mycobacterium tuberculosis* (MTB) and transmitted via respiratory droplets, remains a major global public health problem. The WHO 2024 TB report estimates 10.8 million new cases and 1.25 million deaths globally in 2023, with China identified as a high-burden country [1]. MTB primarily causes pulmonary tuberculosis (PTB) by attacking the lungs, making it one of the deadliest respiratory infections [2]. Crucially, long-term MTB colonization disrupts the lung microecology [3, 4] and reshapes the entire respiratory microbiota [5], processes intrinsically linked to disease pathogenesis and immune modulation.

The respiratory tract functions as a continuous ecosystem, yet current understanding of the TB microbiome is remarkably compartmentalized. While the"lung-gut axis"demonstrates how gut microbiota influences PTB immunity [6, 7] and treatment status [8], the critical interactions within the respiratory tract itself—particularly between the upper and lower compartments—remain poorly explored in TB. This is a significant knowledge gap, as colonization of the upper respiratory tract (especially the oral cavity) is the typical initial step for respiratory pathogens [9]. Oral bacteria are implicated in aspiration pneumonia [10], lung cancer risk [11], and recent evidence even suggests oral microbiota as potential diagnostic biomarkers for PTB [12], underscoring a vital oral-lung connection [13].

While existing microbiome studies in PTB have yielded foundational insights, they exhibit critical limitations. Investigations remain predominantly focused on the bronchoalveolar lavage fluid (BALF) [3, 14, 15] and sputum samples [4, 16], although valuable, this approach provides only a restricted view of lower respiratory tract (LRT) microbiota. Consequently, the potential influence of the upper respiratory tract (URT) microbiome—acting as both a reservoir and gateway for pathogens—on PTB pathogenesis or its utility as a source of biomarkers is largely unaddressed. Furthermore, studies specifically examining microbial interplay, continuity, or correlated dysbiosis between the URT and LRT in PTB patients are notably scarce. Collectively, these gaps result in an absent holistic understanding of the entire respiratory microbiome landscape in PTB.

Therefore, while alterations in the LRT microbiota during PTB are recognized, the impact of PTB on the URT microbiome remains poorly characterized. And the existence of characteristic microbial signatures across the respiratory tract with PTB is unclear. Critically, the nature and extent of microbial interplay between the URT and LRT during active infection are essentially unknown. Addressing these critical gaps is essential to fully elucidate the respiratory microbiome's role in TB pathogenesis, diagnosis, and potential therapy.

To bridge this knowledge gap and overcome the compartmentalized view, we conducted a comprehensive analysis of the entire respiratory microbiome in PTB patients. For the first time, we systematically collected samples from multiple, distinct sites spanning the upper and lower respiratory tract. Using metagenomic sequencing, we characterized site-specific microbial shifts, and most importantly, performed dynamic correlation analyses to uncover the microecological interactions and co-occurrence patterns across the respiratory continuum during active PTB. This multi-site, integrative approach uniquely positions our study to define the pan-respiratory microbiome signature of PTB and reveal previously unexplored interactions.

## Methods

### Participants inclusion criteria

From August 2023 to January 2024, patients undergoing bronchoscopy at Beijing Chest Hospital were screened for inclusion in this study. The individuals who require bronchoscopy include: 1) patients initially presenting with suspected pulmonary tuberculosis requiring bronchoscopic examination to establish a definitive diagnosis, and 2) asymptomatic subjects identified with pulmonary shadowing during routine health check-ups who undergo bronchoscopy for further diagnostic evaluation. The inclusion criteria for the PTB group were as follows: absence of prior TB history, identification of MTB via pathogen detection, initial diagnosis of PTB, and radiological suspicion of PTB without other concurrent pulmonary diseases, and no antibiotic use within two weeks preceding the examination. For the healthy control (HC) group, the criteria included no detection of MTB or common respiratory pathogens through routine pathogen screening, no history of TB, no antibiotics taken within the past two weeks, and lack of common respiratory symptoms such as cough, sputum production, or dyspnea.

### Samples collection

**Fig. 1:**
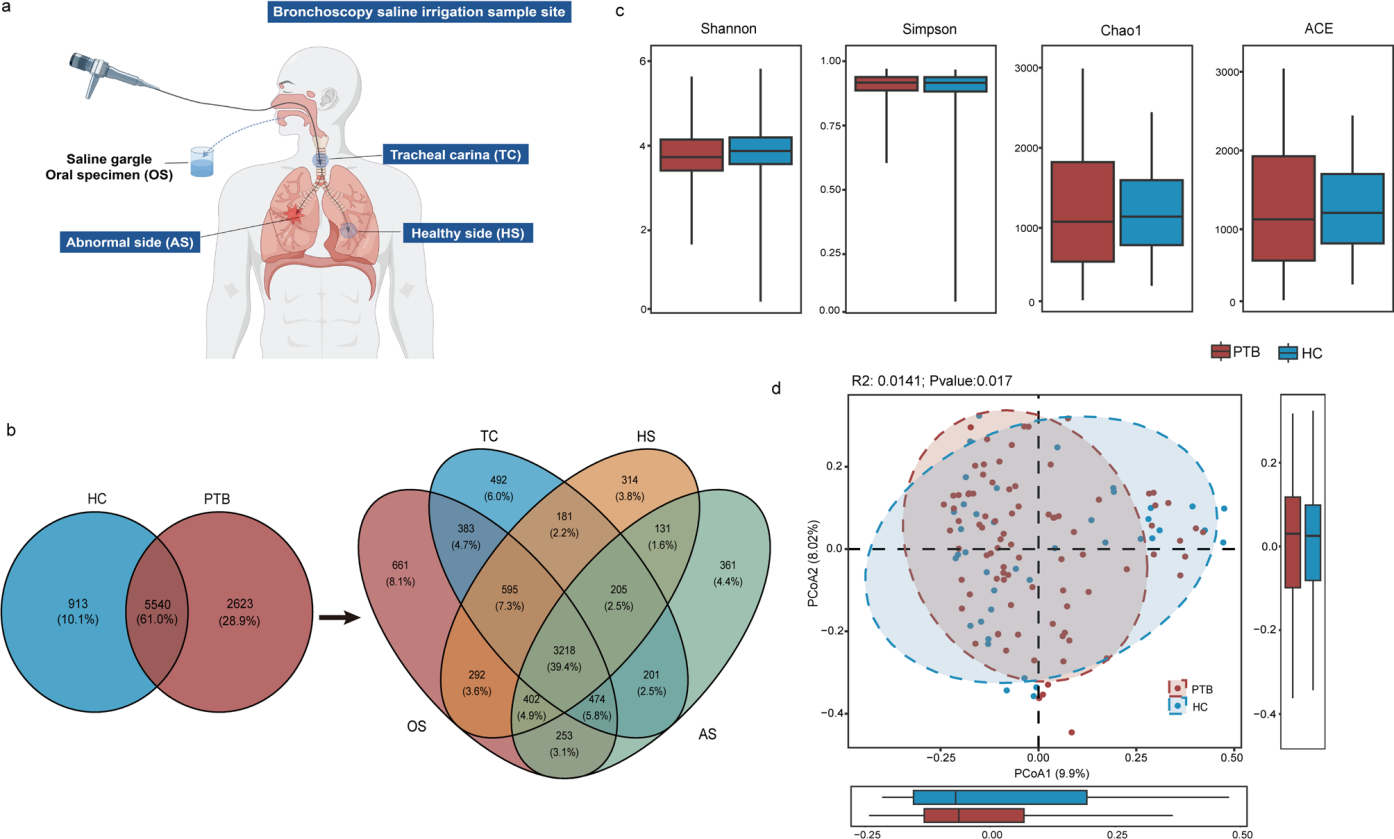
Overall distribution of respiratory tract species and diversity in the two groups. **a** Respiratory tract sampling pattern map. The OS was obtained by gargling. The blue background indicates the location of the lavage obtained by bronchoscopy, namely tracheal carina (TC), healthy side (HS) and affected side (AS) of the lung. **b** The Venn diagram on the left showed the microbial species in the respiratory tract of the HC and PTB groups. The right shows the distribution of species at four sites in the respiratory tract of PTB patients. The rank-sum test analysis of (**c**) alpha diversity and (**d**) β diversity of respiratory microorganisms in PTB patients and HC group was used ADONIS analysis

Respiratory specimens were systematically collected as follows, and the samples are generally collected before lunch in the morning. Oral specimens (OS): individuals were instructed to gargle with 10 mL sterile saline for ≥ 10 s under supervision, then expectorate into a sterile container. The other sample sites were performed standardized sampling [17] via BF-1T260 by professional doctors during bronchoscopy. The bronchoscope is typically introduced via the nasal passage. If nasal insertion is unsuccessful, the oral route is used. First, when the carina is visualized near its superior aspect, 20 mL of sterile saline is instilled. Immediately thereafter, vigorous suction is applied while simultaneously moving the bronchoscope tip in an up-and-down motion to obtain a tracheal carina (TC) sample. This process is applicable to both healthy individuals and PTB patients. Second, for PTB patients, depending on the pulmonary radiography, both the healthy side (HS) and abnormal side (AS) are selected. HS: imaging results of lung generally showed a clear field of vision, or only tiny nodular shadows, and AS: the lung lobe exhibited patchy/cord-like shadows, or tree bud signs, or cavities. Bronchoalveolar lavage fluid (BALF) with 20 mL sterile saline is performed at each site to obtain samples from both lung regions. For HC subjects, we collected BALF from healthy lung lobes with the following criteria: lower lobes with radiologically clear lung fields, or contralateral lower lobes of lung containing incidental micronodules (Fig. 1a). The recovery amount of each site was 5-10 ml. To prevent cross-contamination between sampling sites, sterile single-use collection bottles and tubing were replaced after each site sampling. All bronchoscopes underwent high-level disinfection with 0.55% ortho-phthalaldehyde (Cidex OPA).

### Nucleic acid extraction and metagenomic sequencing

Samples from multiple parts of respiratory tract were frozen at −80℃, and then were sent to Hugo biotech for metagenomic sequencing (Hugo biotech, Beijing, China). Cell-free DNA (cfDNA) [18] was extracted using the QIAamp DNA Micro Kit (QIAGEN, Germany) as instructed. Then, QIAseq™ Ultralow Input Library Kit for Illumina (QIAGEN, Germany) was used to build a DNA library, according to its manual. All constructed libraries were evaluated for quality by Qubit (Thermo Fisher, USA) and Agilent 2100 Bioanalyzer (Agilent Technologies, USA). The qualified DNA libraries were pooled in equal amounts and sequenced on the Illumina Novaseq 6000 platform (Illumina, single-end, 75 bp read length, USA). Each library generated 21.7 million reads on average. Sterile deionized water was extracted alongside the specimens to serve as negative controls (NTC). The metadata can be accessed in https://ngdc.cncb.ac.cn/gsa/browse/CRA019043.

### Metagenomic data analysis

The first critical step in metagenomic analysis is quality control and removal of host contamination from the original reads, Trimmomatic software [19] was used to generate high-quality data by filtering out the splicers, low-quality and shorter sequences. Then KneadData software (https://huttenhower.sph.harvard.edu/kneaddata) was used to remove the human reference database hg38 genome. Bowtie2 [20] software was further used to remove the human NT sequence and YanHuang genome sequences. Kraken2 [21] software was used for taxonomic classification based on the Kraken2 PlusPF database (version 20231009). Kraken2 processed an average of 1.4 sequencing reads without host genome per sample, with 45.7% successfully mapped across microbial taxa. The reads number and reads per million mapped reads (RPM) of each detected microbe was calculated. For microbes with background reads in negative control (NTC), microbes were removed if the RPM ratio defined as RPM_sample_/RPM_NTC_ < 10. For microbes without background reads in negative control, microbes with RPM_sample_ < 0.05 were removed. Microbes with relative abundance higher than 0.01%, sample prevalence coverage above 80% were defined as core microorganisms.

### Neutral model and source tracker analysis

To determine the neutral processes in the lung microbiota of HC individuals and PTB patients, we adopted a custom R script to conduct neutral model, using the OS, HS and AS samples as respective sources of microecological data. The minpack.lm package based on R language was used in nonlinear least square method for parameter fitting. The fitting goodness of the curve was evaluated by the coefficient of determination (*R*^2^). The value ranged from ≤ 0 (no fit) to 1 (perfect fit). 95% confidence interval of Wilson score calculation model prediction based on R package HMisc [22].

SourceTracker [23] predicted the proportion of an unknown experimental sample (sink) arising from known source environments (source) based on Bayesian methods. For each species from sink samples, SourceTracker calculated the possibility scores indicating its potential origin from either the source or the sink. If the source possibility score exceeded the sink score, the species would be regarded as arising from source sample [24]. In this study, OS samples were treated as source, TC, HS, AS samples from the same patient were regarded as sink samples, respectively. We also predicted the HS ratio (source) for OS and TC samples (sink).

### Co-occurrence network analysis

To obtain the stable and steady co-occurrence relationships, genera with average relative abundance ≥ 0.01% were retained to calculate Spearman correlation coefficients across all samples. A correlation was considered statistically robust if Spearman’s correlation coefficient was either ≥ 0.4 or ≤ − 0.4 with significance of *p* < 0.01. Co-occurrence network was visualized in Cytoscape Version 3.9.1 [25].

### Statistical analysis

Alpha diversity was calculated using R package Vegan. The binary Jaccard algorithm calculated the beta diversity distance between samples, followed by principal coordinates analysis (PCoA) using the R package ape. PERMANOVA analysis with default parameters was conducted to compare groups using the adonis2 function in the R package Vegan. The explanatory power (R^2^) of categorical variables was calculated as: ${R}^{2}=\frac{{SS}_{between}}{{SS}_{total}}$, where ${SS}_{between}$ between represents the sum of squares between groups, and ${SS}_{total}$ denotes the total sum of squares calculated from the distance matrix. A higher R^2^ value indicates greater inter-group differences. The STAMP (Statistical Analysis of Metagenomic Profiles) software [26] was used to identify the significant differences in microbial communities. To ensure robustness in the analysis, species with an average relative abundance lower than 0.01% will be excluded. For pairwise comparisons between two groups, the Wilcoxon rank-sum test was employed, while for comparisons among multiple groups, the Kruskal Wallis test was utilized *p* < 0.05 was considered as significant difference.

### Other microbiome datasets collection and processing

Aligned with PTB patient inclusion criteria of the current manuscript, we systematically screened microbiome studies from the past three years, identifying seven relevant publications. Given our investigation of previously unmapped respiratory niches, we carefully benchmarked external upper respiratory tract (URT) samples against our OS and referenced literature BALF datasets to our AS samples. Consequently, all candidate datasets underwent rigorous evaluation for accessibility and analytical utility, culminating in the inclusion of one qualifying BALF dataset [27]. Furthermore, due to unavailable contemporary upper respiratory datasets (2022–2025), we expanded our temporal scope to incorporate a 2018 upper respiratory study [28] with successfully retrieved data, thereby completing our analytical cohort.

The raw sequencing data were downloaded from SRA. The 16S rRNA gene datasets were processed using a standardized pipeline in QIIME 2.0 (v2022.2) [29]. For each dataset, demultiplexed sequencing reads were denoised to generate high quality amplicon sequence variants (ASVs) using the Divisive Amplicon Denoising Algorithm 2 (DADA2) with default parameters. The taxonomy of ASVs was assigned by QIIME2 using a Naive Bayesian Classifier that was pre-trained based on SILVA v13_8 99% OTUs [30]. Subsequently, Mitochondria and chloroplast ASVs were filtered.

## Results

### Demographics of participants and respiratory microbiome overview

A total of 22 PTB patients (88 samples from four sites) and 14 HC individuals (42 samples from three sites) were included in the study, and the two groups were matched by age and sex. Laboratory analyses revealed that white blood cell (WBC) counts and c-reactive protein (CRP) levels were significantly higher in PTB patients than in the HC, indicating an inflammatory response in patients with chronic infection. Other demographic measures showed no significant differences between the two groups (Table 1, Table S1).

**Table 1:** Demographic characteristics and laboratory indexes of enrolled personnel

	PTB (*n* = 22)	HC (*n* = 14)	*P* value
Age (years)	35.36 ± 14.52	36.14 ± 12.40	0.4955
Gender			0.2213
Famle	14	6	-
Male	8	8	-
WBC (× 10^9^/L)	7.19 ± 1.65	6.05 ± 1.24	0.0323
NEU (%)	65.03 ± 7.25	61.90 ± 8.59	0.1941
CRP (≤ 6)	13.38 ± 19.27	1.65 ± 1.26	< 0.0001

*WBC* white blood cell count, *NEU* neutrophil count, *CRP* C-reactive protein

To provide a comprehensive overview of the respiratory microbiome, we conducted a pooled analysis of all samples from each group. In PTB patients, a total of 8,182 microbial species were identified, of which 28.9% were unique to this group. In contrast, the HC group had 6,465 identified species, with 10.1% being unique. Notably, 61% of the microbial species were shared between the two groups (Fig. 1b). Further stratification of PTB patients into TC, HS, OS, and AS revealed distinct microbial profiles. The TC, HS, OS, and AS have 492 (6.0%), 314 (3.8%), 661 (8.1%), and 361 (4.4%) unique species, respectively, with 3218 species (39.4%) shared among all four sites. The pairwise overlaps indicate varying degrees of similarity between groups, such as 181 species (2.2%) shared between TC and HS, 291 (7.3%) between TC and OS, and 292 (3.6%) between OS and HS (Fig. 1b).

Simultaneously, our characterization of the core microbiome demonstrated that 49 (61.2%) of core microbial species were conserved across both PTB and HC cohorts (Fig. S1a). Additionally, comparative assessment of core genomes across distinct respiratory sites demonstrated that 4.5% of core microbial species overlapped among all three sampled sites in the HC group (Fig. S1b), whereas a higher proportion (7.6%) constituted universally shared components across all respiratory sites in the PTB patients (Fig. S1c). Collectively, these results reveal greater microbial diversity and group-unique species in PTB patients compared to HCs, while also indicating that there are overlapping microorganisms at various sites of the respiratory tract.

### Oral microbial diversity of PTB patients is different from that of other parts and HC individuals

To characterize the overall structure of the respiratory microbial ecosystem, we assessed alpha and beta diversity between PTB patients and HC individuals. Alpha diversity analysis revealed distinct patterns: while species evenness and diversity (reflected by Shannon and Simpson indices) showed no significant differences between groups, species richness estimators (Chao1 and ACE indices) conversely indicated significantly higher richness in the PTB group compared to HC (Fig. 1c). Complementing this, PCoA demonstrated significant compositional dissimilarity between PTB and HC microbial communities, highlighting distinct overall community structures (Fig. 1d).

**Fig. 2:**
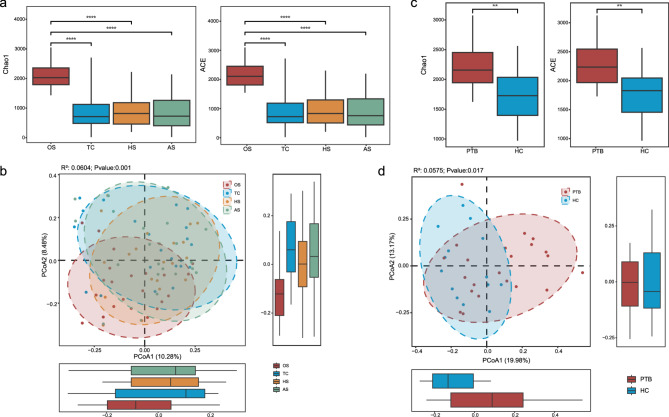
The comparison of microbial diversity between PTB patients and HC group. **a** The alpha diversity results of Chao1 and ACE indices at four sites in PTB patients (OS 22 samples, TC 22, HS 22 and AS 22). **b** The PCoA of four parts with PTB patients. **c** The results of chao1 and ACE index of alpha diversity in OS of PTB group and HC group. **d** Comparison of PCoA in oral samples of PTB group and HC group. (Colors indicate different parts in a-b, and different groups in c-d. Panel (**a**) & (**c**) used rank-sum test, (**b**) & (**d**) used ADONIS analysis, **: *p* < 0.01, ****: *p* < 0.0001)

Then we further analyzed the microbial diversity at different sites to clarify the specific characteristics of the respiratory tract in PTB patients. The Chao1 and ACE indices of the OS were significantly higher than those of other sites, showing striking spatial heterogeneity, whereas TC, HS, and AS exhibited comparable richness without significant inter-site differences (Fig. 2a). The PCoA further revealed that different sites had significant differences in microbiota compositions of microbial community structure, and the R^2^ value explained a small portion of the total variation between groups. Although there is some overlap between oral microorganisms and the other three sites, they can be clearly distinguished, and the communities of the TC, HS, and AS sites have more overlap (Fig. 2b). Similarly, we found the Chao1 and ACE index in OS for the HC individuals are higher than the other sites (Fig. S2e), and PCoA also revealed significant differences in oral cavity (Fig. S2h).

In order to explore the differences in microbial diversity between PTB patients and the HC, we conducted a comparative analysis of different parts of them respectively (in which the AS and HS samples of the PTB group were compared with the HS samples of the HC). We found that the oral Chao1 and ACE indices of PTB group were significantly higher than those in HC individuals (Fig. 2c), and PCoA also showed significant differences in OS microbial community structure between the two groups (Fig. 2d). In contrast, TC sites showed marginally elevated alpha diversity in HC (Fig. S2c) without significant beta-dispersion differences (Fig. S2f). Similarly, lower respiratory sites (AS&HS) revealed no significant alpha (Fig. S2d) or beta diversity distinctions (Fig. S2g) between groups. The outcomes illustrated that the oral microbiota of PTB patients is distinctly different from both other respiratory sites within their own tract and the corresponding site in HC individuals.

### Distribution of microbial species in multiple sites of respiratory tract

**Fig. 3:**
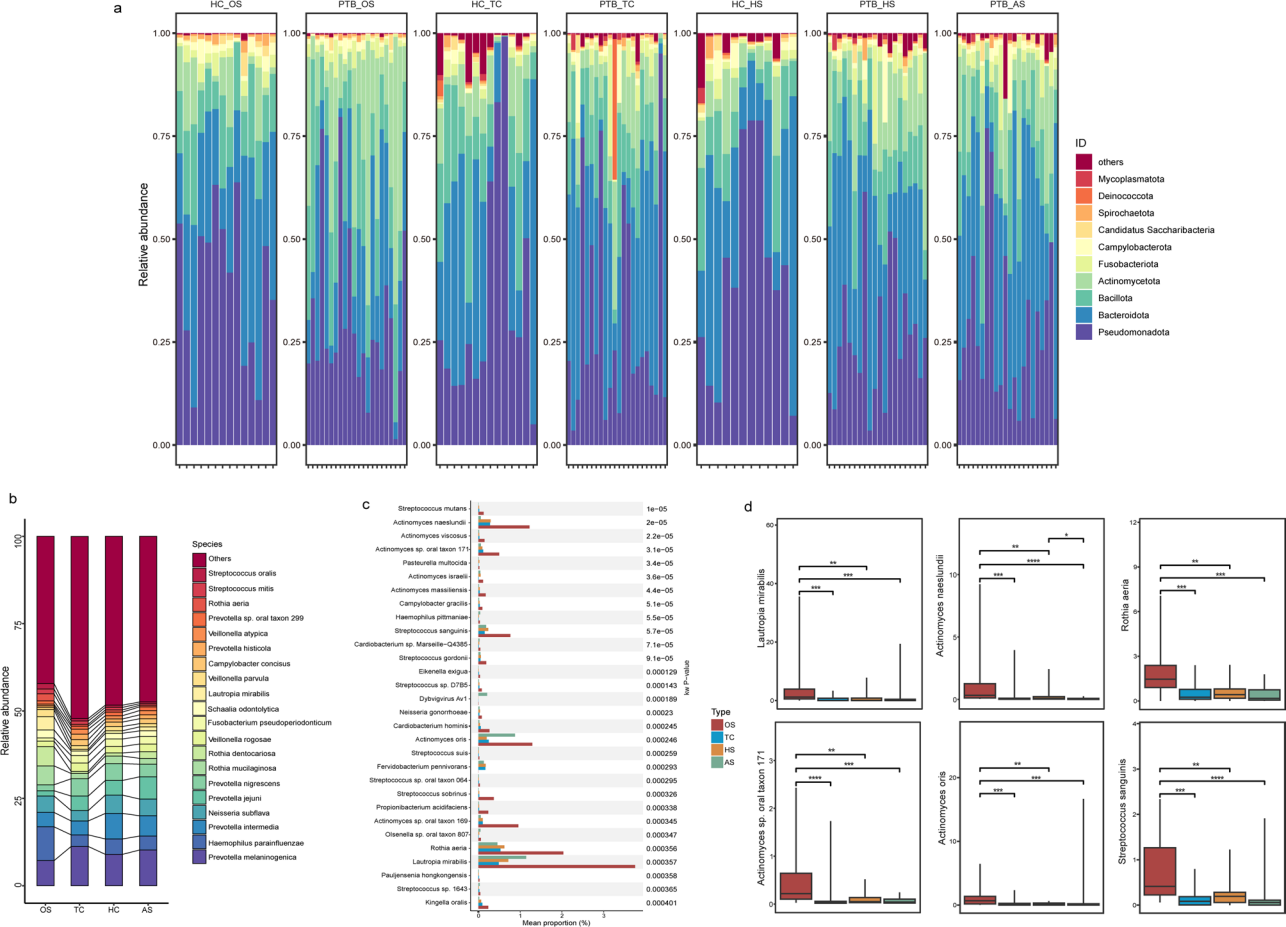
Microbiota composition and distribution of PTB and healthy controls. **a** The phylum level species distribution map of multiple parts between PTB and HC groups. **b** Stack diagram showing the relative abundance of the top 20 species in each sample of PTB patients. **c** Comparative analysis of different species at multiple sites for PTB patients by STAMP analysis (statistical method: KW test). **d** Display of significant distinct species in four respiratory tract sites of PTB patients (Rank-sum test). (*: *p* < 0.05, **: *p* < 0.01, ***: *p* < 0.001, ****: *p* < 0.0001)

To further reveal the features of the respiratory microbiome in PTB patients, we analyzed the specific distribution and differences of microbial species among different sites. At the phylum level, *Proteobacteria*, *Bacteroidota*, *Bacillota*, and *Actinobacteria* collectively represented the dominant taxa in the respiratory microorganism of the respiratory tract sites in PTB and HC groups, comprising the majority of observed species abundance (Fig. 3a). Furthermore, we demonstrated *Prevotella melaninogenica*, *Haemophilus parainfluenzae*, *Prevotella intermedia*, *Neisseria subflava*, and *Prevotella jejuni* emerged as the most abundant species across the core microbiome of PTB and HC individuals (Fig S1d).

Additionally, we quantified the top 20 species of PTB in each part of the respiratory tract. The *Prevotella melaninogenica* maintained high relative abundance, with proportions as follows: OS (7.18%), TC (11.25%), HS (8.99%), and AS (10.23%). Other species with a high prevalence included *Haemophilus parainfluenzae* (OS 9.68%, TC 3.32%, HS 4.4%, AS 4%), *Prevotella intermedia* (OS 4.13%, TC 3.98%, HS 7.32%, AS 5.80%), *Neisseria subflava* (OS 4.7%, TC 2.97%, HS 5.24%, AS 4.77%) and *Prevotella jejuni* (OS 1.48%, TC 4.16%, HS 4.19%, AS 4.77%) (Fig. 3b). This observation shows substantial alignment with the core microbiome species profiling results (Fig S1f). Then, STAMP results identified significant differences in species abundance across sites (Fig. 3c). Specifically, the oral samples demonstrated substantially higher relative abundances of *Lautropia mirabilis*, *Actinomyces naeslundi*, *Rothia aeria*, *Actinomyces sp. oral taxon 171*, *Actinomyces oris* and *Streptococcus sanguinis* compared to the other three respiratory sites (Fig. 3d). To further clarify species distribution patterns in the respiratory tract of PTB patients, we conducted a meta-analysis comparing the top 20 species in our oral samples with corresponding URT datasets from included studies, only “*Prevotella melaninogenica*” was shared among the top 20 oral species across datasets (Table S2). Simultaneously, comparison of our AS samples with BALF specimens from other studies identified four coexisting species, *Prevotella melaninogenica*, *Prevotella jejuni*, *Schaalia odontolytica*, *Campylobacter concisus* (Table S2).

**Fig. 4:**
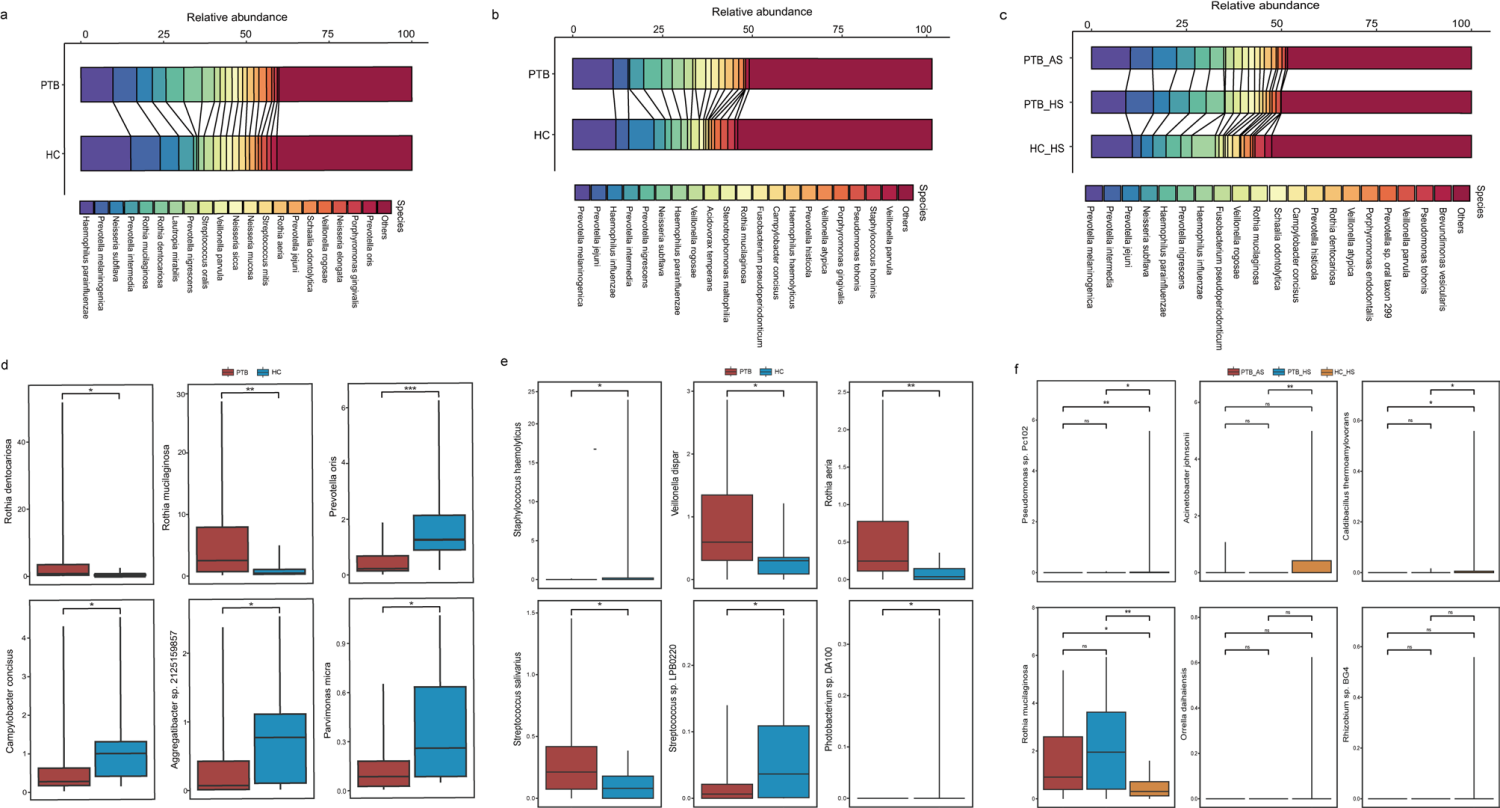
Comparative analysis for respiratory tract sites of microbiota composition and differentially abundant species between PTB and HC groups. The proportion and distribution of top 20 microbial species in the OS (**a**), TC (**b**), lung sites (**c**) of PTB patients and HC group. The box-plot for Rank-sum test statistical results of specific significant differences in the OS (**d**), TC (**e**), lung sites (**f**) between PTB and HC groups. (*: *p* < 0.05, **: *p* < 0.01, ***: *p* < 0.001, ****: *p* < 0.0001)

Next, we compared the distribution of microbial species across different sites in the two groups. Among the top 20 identified species of OS, *Rothia mucilaginosa* (5.4% vs 0.95%) and *Rothia dentocariosa* (5.5% vs 0.52%) showed higher relative abundance in PTB group compared to the HC (Fig. 4a). Further analysis of species differences revealed that in the OS, *R. dentocariosa*, *R. mucilaginosa* and *Prevotella oris* were significantly more abundant in PTB patients, whereas *Campylobacter concisus* was more abundant in the HC group (Fig. 4d & S3a). Additionally, in the TC site samples, the top 20 species analysis identified the presence of *Stenotrophomonas maltophilia* in PTB patients, a species that was not detected in the HC group (Fig. 4b). And comparative results also showed that *Veillonella dispar*, *Rothia aeria*, and *Streptococcus salivarius* were significantly more prevalent in PTB patients (Fig. 4e & S3b). Finally, species abundance analysis in lung samples revealed that *Prevotella intermedia* was more abundant in the PTB group (PTB_AS 5.81%, PTB_HS 7.32%, HC_HS 2.32%) (Fig. 4c), and statistical analysis further showed that the abundance of *Rothia mucilaginosa* was significantly higher in PTB patients (Fig. 4f & S3c). Concurrently, we found that the levels of *Haemophilus parainfluenzae*, *Neisseria elongata*/*sicca* and *Campylobacter rectus* were significantly higher for the oral cavity compared to the other two sites in HC individuals (Fig. S4b-c).

### Disturbance of respiratory tract flora in PTB patients promotes the migration of upper and lower respiratory tract microorganisms

**Fig. 5:**
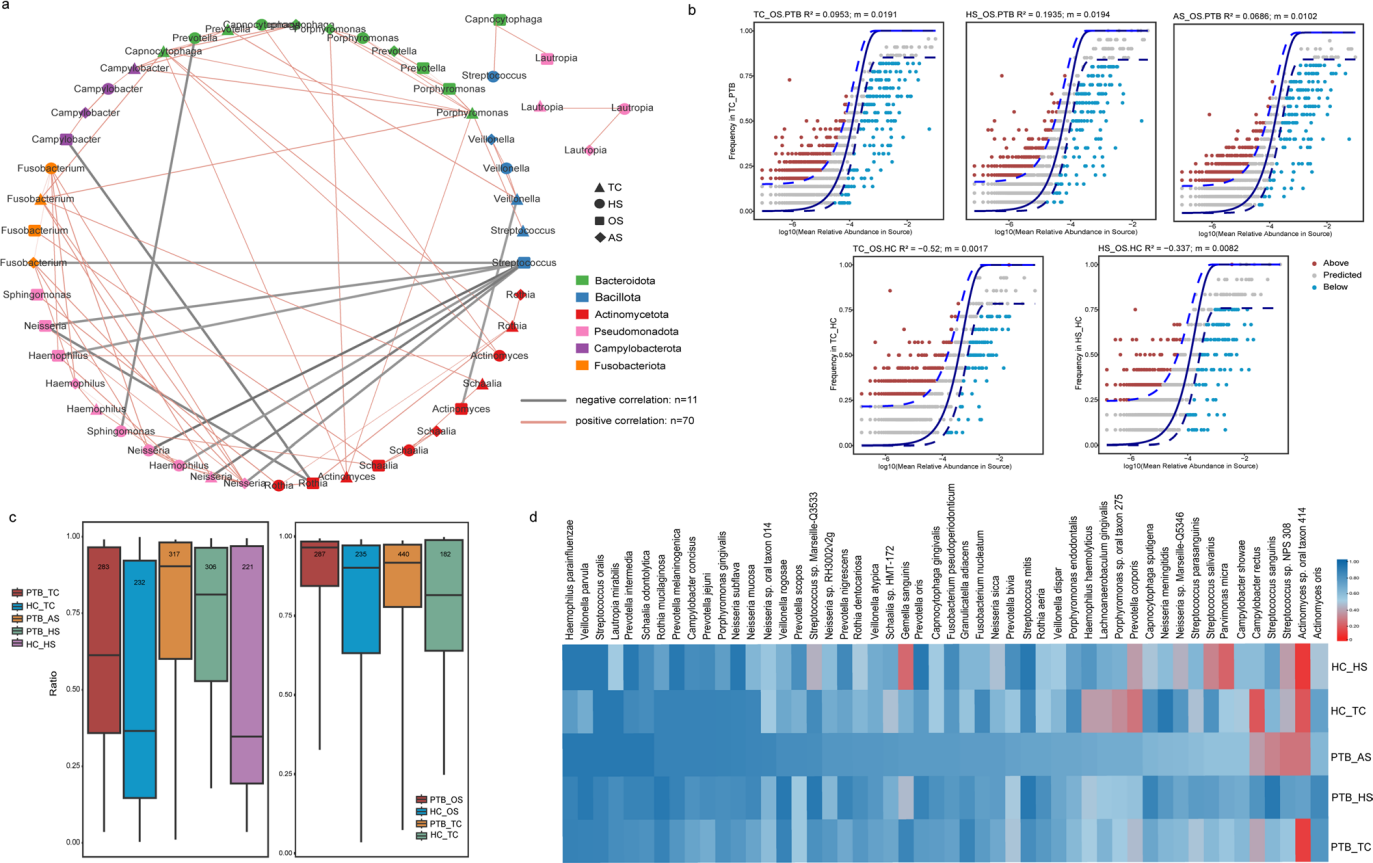
Interaction network of microorganisms in respiratory tract of patients with PTB and changes among different parts. **a** The collinear network of genus levels among the four sites of PTB patients. Different shapes representing different sites, the red line showing positive correlation and the gray line showing negative correlation. **b** The neutral model for fitting each part of PTB patients and HC group based on oral samples, R^2^ represents the degree of fitting, and the larger the value, the better the fitting. **c** Based on oral cavity (right) and HS (left) samples, SourceTracker analysis was performed on each site, and the proportion of oral microorganisms in each site of PTB and HC groups was showed (Rank sum test). The numbers in the box-plot represent the number of migrating species. **d** The distribution of species with high migration frequency (≥ 75%) from OS in the three parts of PTB group, and their corresponding frequency in HC group were shown by heat map. The frequency was indicated by the color depth

Based on the analysis of the above differential features, we speculated that in the chronic infection state of PTB patients, the mutual flow of microorganisms between the upper and lower respiratory tracts may influence the dynamic changes in the overall microbiome. To test this hypothesis, we performed a co-occurrence network analysis of microorganisms from multiple sites in PTB patients. The results showed that there were interactions among the four sites—OS, TC, HS and AS—with microbial species primarily exhibiting positive correlations, suggesting potential cooperative relationships under certain conditions. The fewer negative correlations indicated less competition or exclusion, or these may be limited to specific microbes (Fig. 5a). Further, we found that the oral *Streptococcus* had a strong negative interaction with other genera. And *Neisseria* also showed strong positive correlation across all sites. Additionally, *Fusobacterium*, *Prevotella* and others all exhibited different interactions among different parts (Fig. 5a).

To assess the randomness in species distribution across these communities, we fitted a neutral model to the different respiratory sites. The results indicated that the neutral model fits well for the PTB group, suggesting that species distribution in these communities is likely driven by random migration. In contrast, the *R*^2^ value for the HC group was negative, indicating that the neutral model does not adequately explain species distribution in these communities (Fig. 5b).

In order to investigate microbial migration between the upper and lower respiratory tracts, we used SourceTracker to conduct traceability analysis. Meanwhile, we used the data obtained from SourceTracker analysis to conduct quantitative statistics on the number of transferred species, in order to roughly determine how many species have been moved. First, we used the OS samples as a baseline to track its migration to the TC, HS, and AS sites. The PTB group revealed 283 species transferred from OS to TC, 317 to AS, and 306 to HS, and the HC group exhibited 232 species migrating from oral sources to TC and 221 to HS. Although the proportion of oral microbiota present in various respiratory sites of PTB patients did not reach statistical significance, there was a trend toward an increase compared to the HC group. Due to the lack of AS samples from HC individuals, we also analyzed the bacterial migration based on the HS of lung. Similarly, we found that the number of pulmonary species flowing to the other two respiratory parts in PTB patients was higher than that in the HC (Fig. 5c, Table S3). Lastly, we conducted a quantitative analysis of the high-frequency migration species (contaminate/exists, Table S3). We screened for bacteria from the OS that migrated to the TC, HS and AS sites with a frequency of more than 75% in PTB patients and calculated the migration frequency of these bacteria in the HC group. The data revealed that the migration frequency of the bacteria with high-frequency bacteria in PTB patients was relatively low in the HC (Fig. 5d).

## Discussion

Microbial communities exhibit inherent dynamism, undergoing continuous compositional fluctuations [31]. While maintaining resilient homeostasis under healthy conditions, disease states provoke dysregulation of community structure—progressing to pathological dysbiosis that exacerbates clinical progression. Our study delivers unprecedented view of this process in pulmonary tuberculosis through comprehensive profiling of: (1) spatial microbial distribution across respiratory niches, and (2) inter-site translocation dynamics within the respiratory tract.

The significantly elevated WBC counts and CRP levels in PTB patients (Table 1) confirm a systemic inflammatory state, consistent with chronic MTB infection. Our metagenomic profiling revealed a substantially expanded respiratory microbiome in PTB patients compared to HC (8,182 vs. 6,465 species, Fig. 1b), consistent with previous reports [31]. This suggests that the chronic inflammatory milieu in PTB may foster niche diversification, potentially enabling the colonization of opportunistic or inflammation-adapted taxa absent in healthy states. Notably, while a core set of microbial species (61.2%) was conserved across both groups – indicating shared foundational communities – the increased uniqueness in PTB points to disease-specific microbial recruitment or expansion (Fig. S1a).

Despite comparable α-diversity, PCoA revealed distinct microbiota structures in PTB patients versus HCs, confirming disease-associated restructuring. This shift was most pronounced orally (Fig. 2a), where significantly higher diversity in PTB patients mirrors recent findings [12]. And the OS core microbiome harbored the highest number of unique species (Fig. S1c), potentially reflecting its role as the primary gateway and a diverse ecological reservoir. While lower respiratory sites (TC/HS/AS) showed minimal α/β-diversity differences between PTB and HC (Fig. S2c, d, g & h). Among them, the analysis of lung diversity in PTB patients was consistent with previous studies showing no significant differences [32, 33], but contrasting with others reporting alterations [34]. This suggests that there is little consensus among existing studies on lung microbial diversity [35].

The oral cavity serves as the primary gateway for microbial ingress into the LRT, with its microbiota vulnerable to dysregulation through TB-induced alterations in the local immune microenvironment [36, 37]. Concurrently, immunosuppression impairs the barrier function of the oral mucosa, while heightened inflammatory responses compromise mucosal integrity—creating favorable conditions for microbial adhesion and colonization [38]. This mechanistic framework explains although the overall diversity has not changed significantly, the significantly elevated Chao1 and ACE indices in PTB patients versus HC (Fig. 2c) and unchanged Shannon&Simpson indices (Fig. S2b). This study further demonstrates that PTB disease reshapes microbial membership while maintaining ecosystem architecture stability, aligning with previous research findings [34]. Although PTB alters microbiota composition in both oral and pulmonary niches, TC as the principal airway bifurcation point, likely maintains a relatively stable core microbiota, potentially safeguarded by local mucosal immunity and ciliary clearance mechanisms [39].

Consistent with previous studies, the *Proteobacteria*, *Bacteroidota*, *Bacillota*, and *Actinobacteria* these phyla were dominant in both the PTB and HC group [13, 32, 36] (Fig. 3a). Critically, we identified distinct oral microbial signatures in PTB patients, including *Lautropia mirabilis* that associated with invasive manifestations [40, 41]. In addition, *Actinomyces naeslundii*, *Actinomyces sp. oral taxon 171*, and *Actinomyces oris* were often implicated in oral diseases like pulpitis, and were considered opportunistic pathogens [42, 43]. While typically commensal, *Rothia aeria* demonstrates pathogenic potential in periodontal, necessitating clinical monitoring when detected in immunodeficient individuals [44, 45]. *Streptococcus sanguinis* is an oral symbionte and is widely present in the oral cavity, but it has the ability to antagonize oral pathogens [46]. Thus, these species significantly diverged from other respiratory sites, suggesting their role in driving dysbiosis dynamics.

Comparative analysis across respiratory sites revealed PTB-specific pathobiont enrichment where oral *R. mucilaginosa*, *R. dentocariosa*, and *P. oris* showed significant elevation in PTB patients (Fig. 4d). This pattern extended to lung samples for *R. mucilaginosa* (Fig. 4d) and tracheal carina for *R. aeria* (Fig. 4b). These findings align with established disease associations: although *Rothia* species produce pathogen-inhibiting enzymes [47], they paradoxically exacerbate respiratory disease progression and demonstrate MTB co-occurrence [33, 48]. Similarly, *P. oris* displays empyema [49] and bacteremia [50] risks. Additionally, consistent with existing literature [51], *Haemophilus parainfluenzae* demonstrates significantly greater oral abundance in HC than PTB patients (Fig. 4a). While maintaining commensal dominance in homeostasis, retaining potential for opportunistic pathogenesis under immunosuppression [52]. Simultaneously, tracheal dysbiosis featured both *Streptococcus salivariu*s and increased *R. aeria*, those linked to pulmonary hypertension [53] and inhibited pathogenic bacterial growth [47]. Finally, in the analysis of lung samples, *Prevotella intermedia* exhibited higher abundance in PTB patients than in the HC. Previous research has indicated different subspecies of *Prevotella intermedia* can exert varying degrees of virulence [54]. Collectively, this multi-site pathobiont expansion underscores the oral microbiota's pathogenic potential in PTB through translocation and virulence expression.

Our integrative analysis reveals that PTB patients destabilizes the respiratory ecosystem through inflammation-driven loss of niche compartmentalization. The well fit of the neutral model in PTB patients (R^2^ >0, Fig. 5b) demonstrates stochastic migration dominates community assembly, contrasting sharply with deterministic structuring in HC. This breakdown stems from chronic MTB infection impairing mucociliary clearance and epithelial integrity, sustained inflammation degrading anatomical barriers that normally restrict microbial translocation, and systemic immunosuppression disabling site-specific immune surveillance—collectively eroding respiratory niche boundaries.

Microbial co-occurrence networks in PTB patients revealed predominantly cooperative interactions, reflecting pathobiont synergy (Fig. 5a). Commensal *Neisseria* species, as typically benign colonizers of oral/nasal niches [55], exhibited extensive positive correlations. Furthermore, *Fusobacterium* demonstrated synergistic pathogenicity with respiratory pathogens [56]. And *Prevotella* can also spread through the gastrointestinal tract and respiratory tract and interact with the flora at different sites to affect disease [57]. The competitive interaction analyses suggest that PTB disease fosters an environment conducive to collaborative microbial resource competition, thereby enabling polymicrobial advancement across respiratory niches.

Finally, the analysis results of SourceTracker indicated that the number of species migrations from the mouth to the LRT in PTB is slightly higher than that in HC (Fig. 5c), which may be related to the increased diversity of oral microorganism (Fig. 2a&c). Subsequently, the high-frequency transfer species we obtained through frequency calculation may drive changes in the entire respiratory tract microbiota and may have an impact on PTB disease. Notably, *Veillonella parvula*, was known to migrate to the gut and exacerbate inflammatory processes [58], that may be the pathogenic translocation dynamics. Simultaneously, *R. mucilaginosa* demonstrated both statistical significance in differential abundance analysis (Fig. 4d & f) and high-frequency translocation patterns (Fig. 5d), suggesting its critical involvement in PTB pathogenesis [44, 47, 48]. Furthermore, *Prevotella intermedia*'s previously documented virulence mechanisms imply adverse impacts on disease progression [54]. Overall, these species exemplify microbiota-pathogen interactions warranting mechanistic investigation in tuberculosis.

We acknowledge that this study has several limitations. First, the modest cohort size and single-center design may constrain population generalizability. Secondly, the absence of orthogonal validation for metagenomically-identified differential species constitutes a methodological limitation, stemming from biospecimen constraints. To address this, we have subsequent related research plans to further explore the functional roles of these differential species. Moreover, the limited number of datasets included in this meta-analysis, combined with the scarcity of conserved species observed, may be attributable to regional variations, individual heterogeneity, and methodological discrepancies in sampling. These factors necessitate validation through expanded datasets in future studies. Finally, while 75 bp single-end sequencing enabled taxonomic profiling, its limited resolution for strain-level analysis suggests future work could employ long-read platforms.

## Conclusion

This study provides the first comprehensive analysis of the microbiome across multiple respiratory tract sites in patients with PTB, revealing key microecological characteristics associated with the disease. Compared with HC individuals, the *Rothia*, *Prevotella* and *Actinomyces* exhibited higher proportion in PTB. And the microbial characteristics in the oral site were distinct from those in other sites and in HC individuals, especially the high frequency of *Rothia mucilaginosa* in oral site. Furthermore, *Streptococcus*, *Neisseria*, *Prevotella* and *Fusobacterium* have strong interactions with other species at various respiratory sites of PTB patients, as well as frequent communication between these species during migration in the upper and lower respiratory tracts. These findings enhance our understanding of PTB mechanisms and their effects on disease progression and treatment, particularly concerning the emergence of distinct species, which may aid in developing new microbial intervention strategies.

## Supplementary Information

Supplementary Material 1: Fig. S1 Core microbiota & dominant species across respiratory sites in PTB and HC. (a) The core microbiome species between PTB and HC in all respiratory sites. (b) The core microbiome species of three sites in HC group. (c) The core microbiome species of OS, TC, HS, AS with PTB. (d) Top 20 species of core microbiota between PTB and HC. (e) Top 20 species of core microbiota of OS, TC, HS in the HC. (f) Top 20 species of core microbiota of four sites with PTB. Fig. S2 The comparison of microbial diversity between PTB patients and HC group. (a) The Shannon and Simpson indices of OS, TC, HS, AS with PTB. (b) The Shannon and Simpson indices of OS between PTB and HC. (c) Alpha diversity results of TC sample between PTB and the HC. (d) Alpha diversity of lung samples between PTB patients and HC. (e) Alpha diversity of OS, TC, HS of HC individuals. (f) The beta diversity (PCoA) of oral samples between PTB patients and HC. (g) The PCoA result of lungs samples in PTB and HC group. (h) PCoA result of all respiratory tract sites of HC individuals. (*:*p* < 0.05, **: *p* < 0.01, ns: no significance). Fig. S3 The STAMP results between PTB and the HC. (a) The comparative analysis of oral sites by Wilcoxon rank-sum test of STAMP between PTB and the HC. (b) STAMP results of TC samples between PTB and the HC. (c) STAMP results of lung samples between PTB and the HC. Fig. S4 Respiratory microbiota in HC: site-specific composition and distribution. (a) Common and specific species of Venn diagrams of in each part in the HC group. (b) Stack diagram showing the relative abundance of the top 20 species in each sample of PTB patients. (c) STAMP results of OS, TC, HS samples in HC individuals (statistical method: KW test).

Supplementary Material 2.

## Data Availability

Sequence data that support the findings of this study have been deposited in in the China National Center for Bioinformation repository with the primary accession code PRJCA030141.

## Abbreviations

TB: Tuberculosis
MTB: Mycobacterium tuberculosis
PTB: Pulmonary tuberculosis
HC: Healthy control
OS: Oral specimens
TC: Tracheal carina
HS: Healthy side
AS: Abnormal side
BALF: Bronchoalveolar lavage fluid
PCoA: Principal coordinates analysis
STAMP: Statistical Analysis of Metagenomic Profiles
WBC: White blood cell
CRP: C-reactive protein
LRT: Lower respiratory tract
URT: Upper respiratory tract
