# Supplementary figures and images for: Dysbiosis associated with enhanced microbial mobility across the respiratory tract in pulmonary tuberculosis patients
Source: BMC Microbiol. 2025 Aug 12;25:499. doi: 10.1186/s12866-025-04206-x (PMC12341359; doi:10.1186/s12866-025-04206-x)

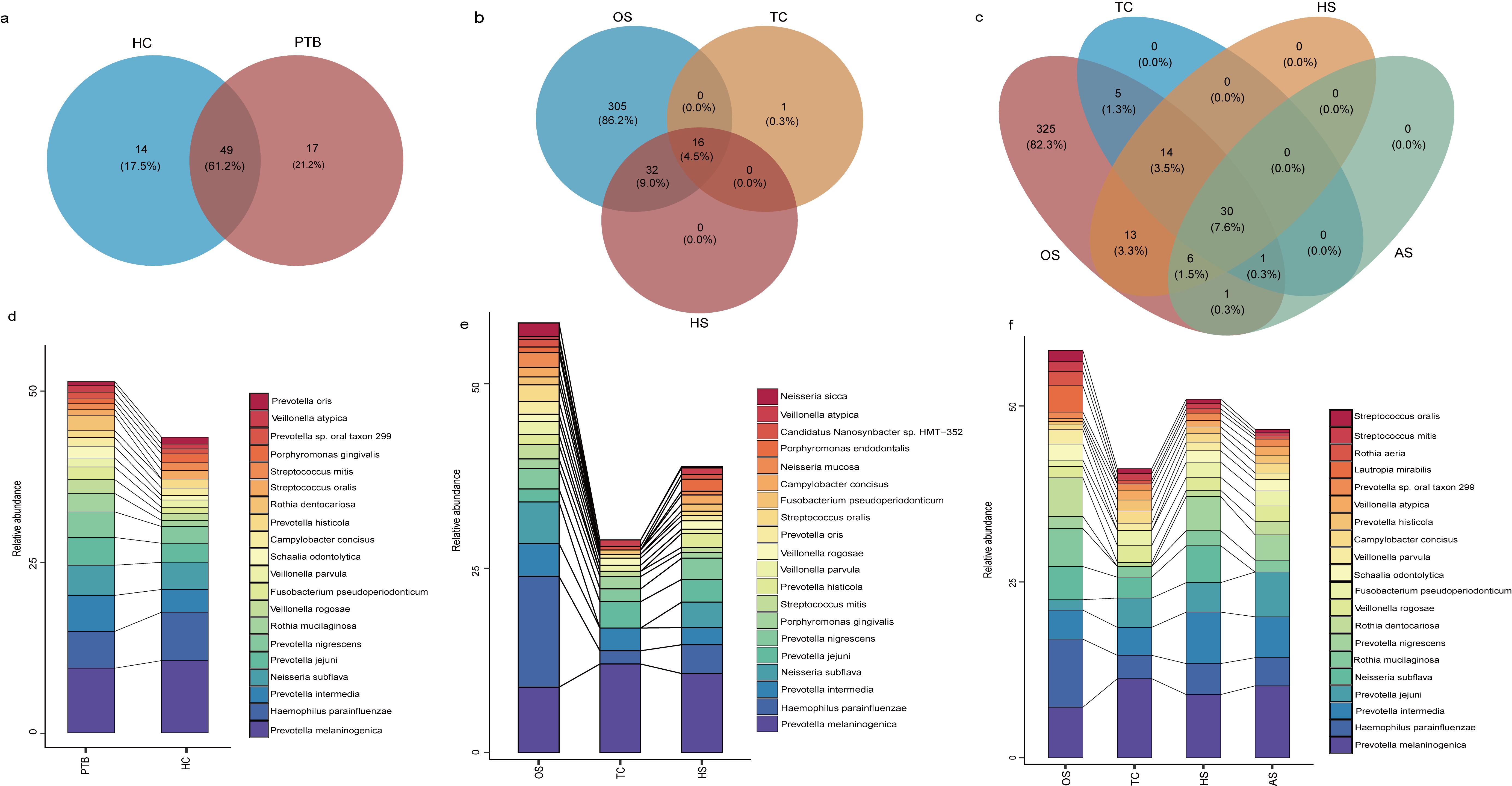

Supplement: Supplementary file 1 — Supplementary Material 1: Fig. S1 Core microbiota & dominant species across respiratory sites in PTB and HC. (a) The core microbiome species between PTB and HC in all respiratory sites. (b) The core microbiome species of three sites in HC group. (c) The core microbiome species of OS, TC, HS, AS with PTB. (d) Top 20 species of core microbiota between PTB and HC. (e) Top 20 species of core microbiota of OS, TC, HS in the HC. (f) Top 20 species of core microbiota of four sites with PTB. Fig. S2 The comparison of microbial diversity between PTB patients and HC group. (a) The Shannon and Simpson indices of OS, TC, HS, AS with PTB. (b) The Shannon and Simpson indices of OS between PTB and HC. (c) Alpha diversity results of TC sample between PTB and the HC. (d) Alpha diversity of lung samples between PTB patients and HC. (e) Alpha diversity of OS, TC, HS of HC individuals. (f) The beta diversity (PCoA) of oral samples between PTB patients and HC. (g) The PCoA result of lungs samples in PTB and HC group. (h) PCoA result of all respiratory tract sites of HC individuals. (*:p < 0.05, **: p < 0.01, ns: no significance). Fig. S3 The STAMP results between PTB and the HC. (a) The comparative analysis of oral sites by Wilcoxon rank-sum test of STAMP between PTB and the HC. (b) STAMP results of TC samples between PTB and the HC. (c) STAMP results of lung samples between PTB and the HC. Fig. S4 Respiratory microbiota in HC: site-specific composition and distribution. (a) Common and specific species of Venn diagrams of in each part in the HC group. (b) Stack diagram showing the relative abundance of the top 20 species in each sample of PTB patients. (c) STAMP results of OS, TC, HS samples in HC individuals (statistical method: KW test). [file 12866_2025_4206_MOESM1_ESM.zip › Fig S1-4/Fig S1.tif]

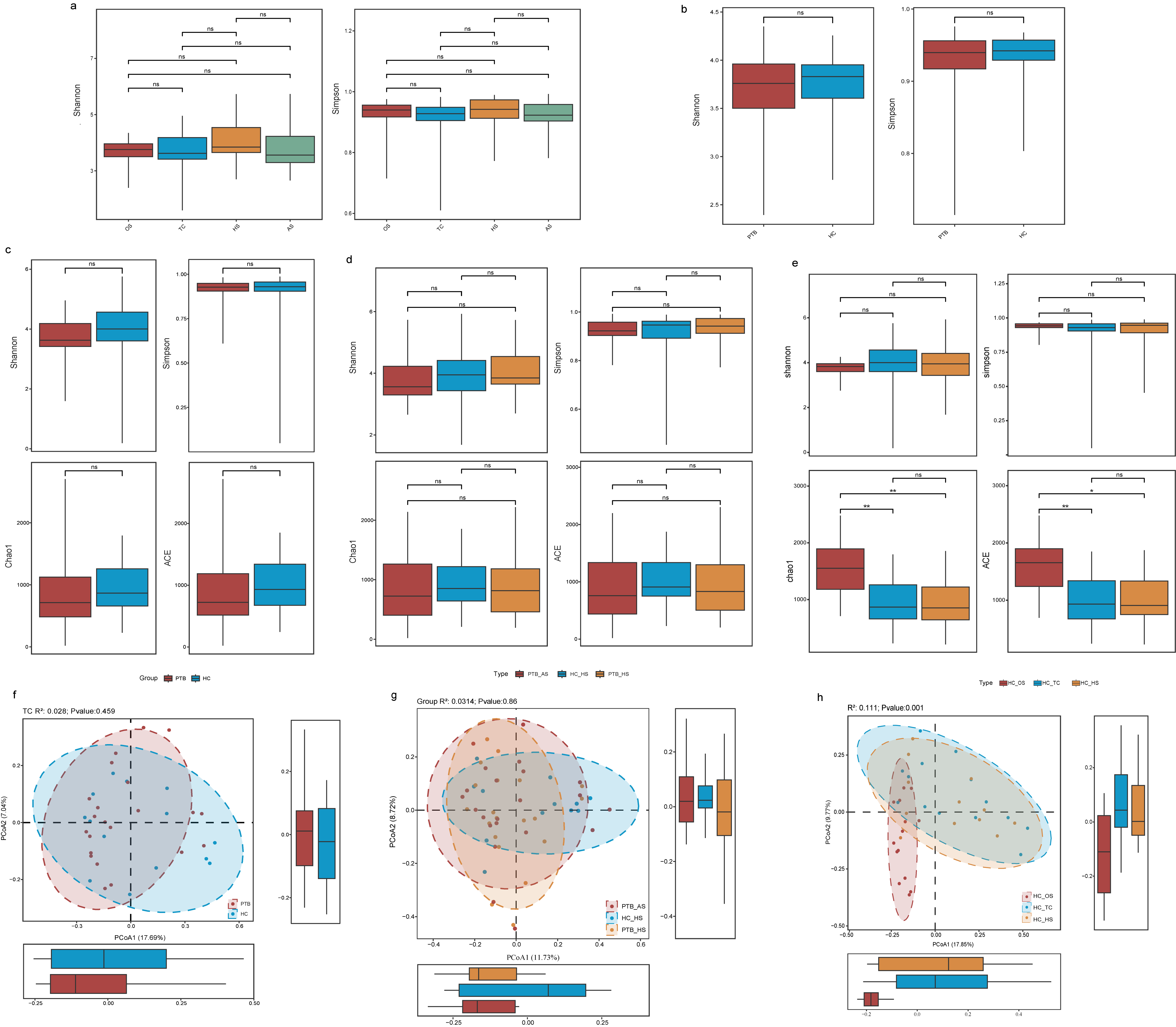

Supplement: Supplementary file 1 — Supplementary Material 1: Fig. S1 Core microbiota & dominant species across respiratory sites in PTB and HC. (a) The core microbiome species between PTB and HC in all respiratory sites. (b) The core microbiome species of three sites in HC group. (c) The core microbiome species of OS, TC, HS, AS with PTB. (d) Top 20 species of core microbiota between PTB and HC. (e) Top 20 species of core microbiota of OS, TC, HS in the HC. (f) Top 20 species of core microbiota of four sites with PTB. Fig. S2 The comparison of microbial diversity between PTB patients and HC group. (a) The Shannon and Simpson indices of OS, TC, HS, AS with PTB. (b) The Shannon and Simpson indices of OS between PTB and HC. (c) Alpha diversity results of TC sample between PTB and the HC. (d) Alpha diversity of lung samples between PTB patients and HC. (e) Alpha diversity of OS, TC, HS of HC individuals. (f) The beta diversity (PCoA) of oral samples between PTB patients and HC. (g) The PCoA result of lungs samples in PTB and HC group. (h) PCoA result of all respiratory tract sites of HC individuals. (*:p < 0.05, **: p < 0.01, ns: no significance). Fig. S3 The STAMP results between PTB and the HC. (a) The comparative analysis of oral sites by Wilcoxon rank-sum test of STAMP between PTB and the HC. (b) STAMP results of TC samples between PTB and the HC. (c) STAMP results of lung samples between PTB and the HC. Fig. S4 Respiratory microbiota in HC: site-specific composition and distribution. (a) Common and specific species of Venn diagrams of in each part in the HC group. (b) Stack diagram showing the relative abundance of the top 20 species in each sample of PTB patients. (c) STAMP results of OS, TC, HS samples in HC individuals (statistical method: KW test). [file 12866_2025_4206_MOESM1_ESM.zip › Fig S1-4/Fig S2.tif]

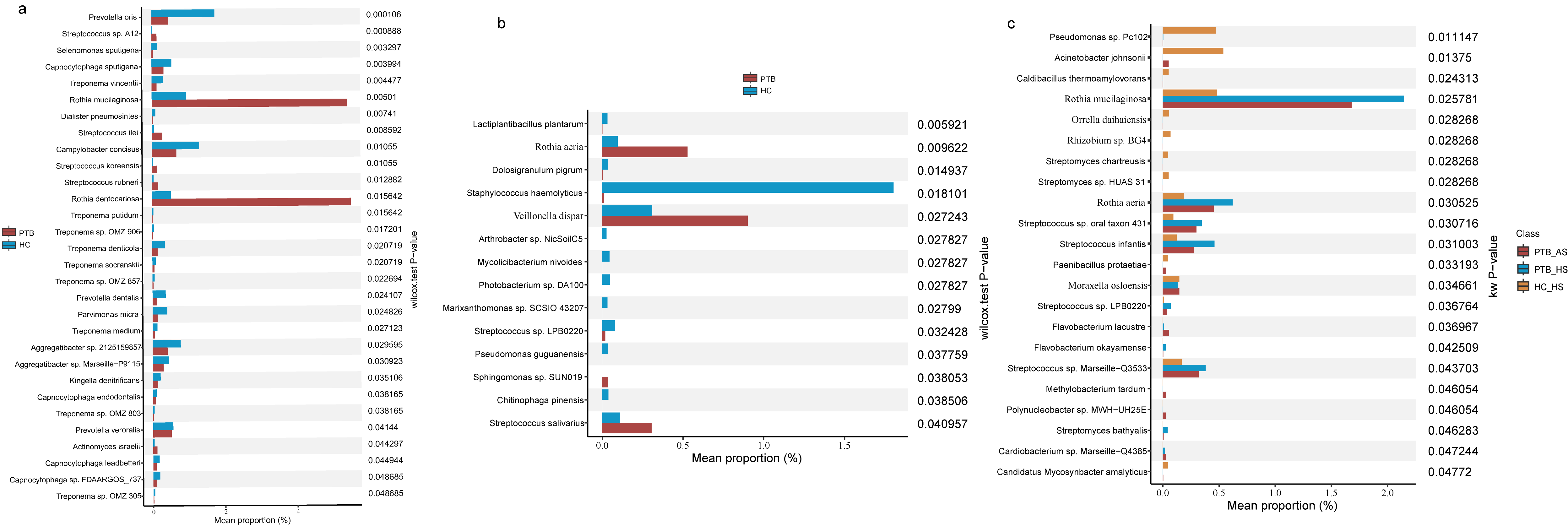

Supplement: Supplementary file 1 — Supplementary Material 1: Fig. S1 Core microbiota & dominant species across respiratory sites in PTB and HC. (a) The core microbiome species between PTB and HC in all respiratory sites. (b) The core microbiome species of three sites in HC group. (c) The core microbiome species of OS, TC, HS, AS with PTB. (d) Top 20 species of core microbiota between PTB and HC. (e) Top 20 species of core microbiota of OS, TC, HS in the HC. (f) Top 20 species of core microbiota of four sites with PTB. Fig. S2 The comparison of microbial diversity between PTB patients and HC group. (a) The Shannon and Simpson indices of OS, TC, HS, AS with PTB. (b) The Shannon and Simpson indices of OS between PTB and HC. (c) Alpha diversity results of TC sample between PTB and the HC. (d) Alpha diversity of lung samples between PTB patients and HC. (e) Alpha diversity of OS, TC, HS of HC individuals. (f) The beta diversity (PCoA) of oral samples between PTB patients and HC. (g) The PCoA result of lungs samples in PTB and HC group. (h) PCoA result of all respiratory tract sites of HC individuals. (*:p < 0.05, **: p < 0.01, ns: no significance). Fig. S3 The STAMP results between PTB and the HC. (a) The comparative analysis of oral sites by Wilcoxon rank-sum test of STAMP between PTB and the HC. (b) STAMP results of TC samples between PTB and the HC. (c) STAMP results of lung samples between PTB and the HC. Fig. S4 Respiratory microbiota in HC: site-specific composition and distribution. (a) Common and specific species of Venn diagrams of in each part in the HC group. (b) Stack diagram showing the relative abundance of the top 20 species in each sample of PTB patients. (c) STAMP results of OS, TC, HS samples in HC individuals (statistical method: KW test). [file 12866_2025_4206_MOESM1_ESM.zip › Fig S1-4/Fig S3.tif]

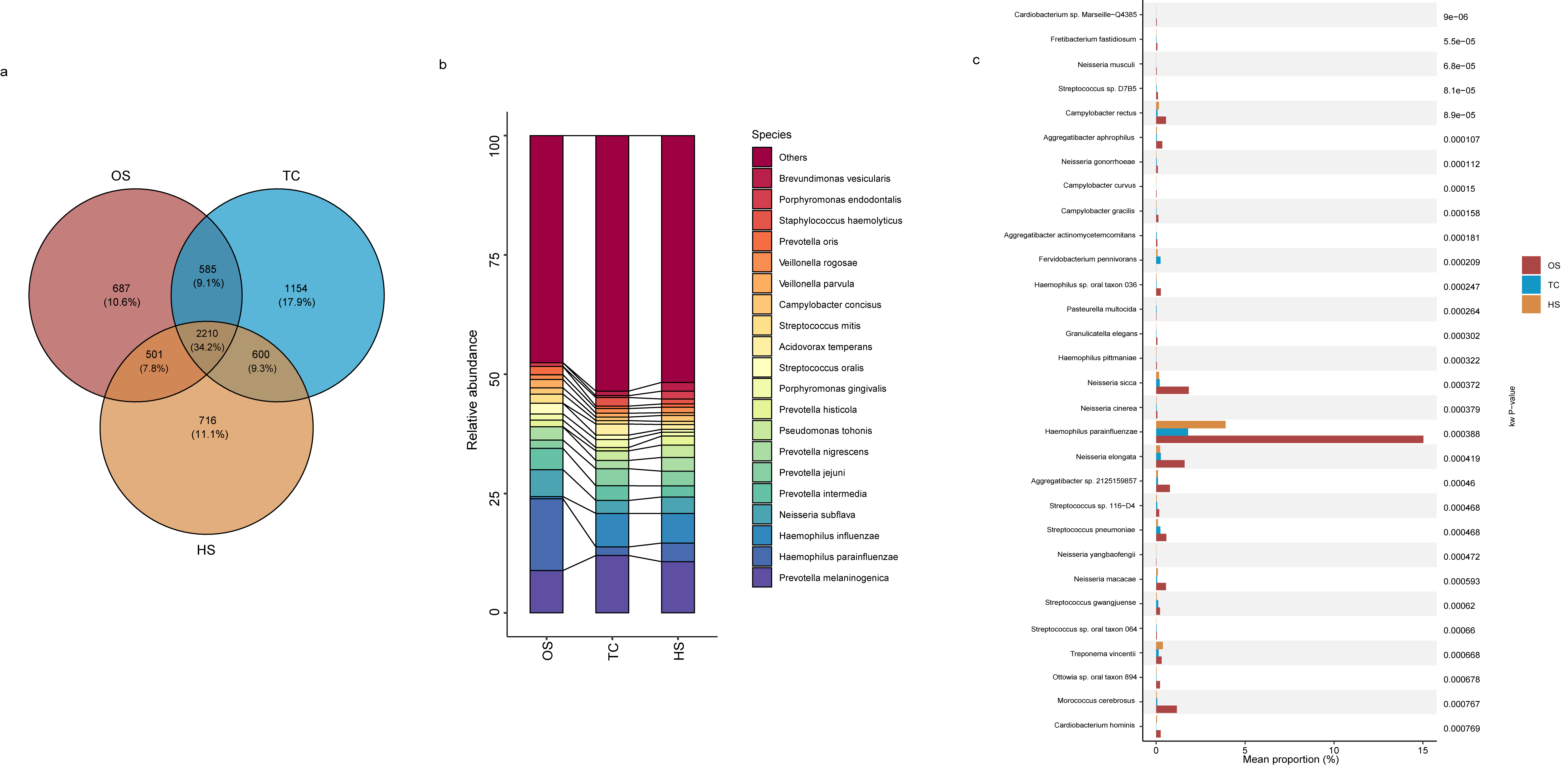

Supplement: Supplementary file 1 — Supplementary Material 1: Fig. S1 Core microbiota & dominant species across respiratory sites in PTB and HC. (a) The core microbiome species between PTB and HC in all respiratory sites. (b) The core microbiome species of three sites in HC group. (c) The core microbiome species of OS, TC, HS, AS with PTB. (d) Top 20 species of core microbiota between PTB and HC. (e) Top 20 species of core microbiota of OS, TC, HS in the HC. (f) Top 20 species of core microbiota of four sites with PTB. Fig. S2 The comparison of microbial diversity between PTB patients and HC group. (a) The Shannon and Simpson indices of OS, TC, HS, AS with PTB. (b) The Shannon and Simpson indices of OS between PTB and HC. (c) Alpha diversity results of TC sample between PTB and the HC. (d) Alpha diversity of lung samples between PTB patients and HC. (e) Alpha diversity of OS, TC, HS of HC individuals. (f) The beta diversity (PCoA) of oral samples between PTB patients and HC. (g) The PCoA result of lungs samples in PTB and HC group. (h) PCoA result of all respiratory tract sites of HC individuals. (*:p < 0.05, **: p < 0.01, ns: no significance). Fig. S3 The STAMP results between PTB and the HC. (a) The comparative analysis of oral sites by Wilcoxon rank-sum test of STAMP between PTB and the HC. (b) STAMP results of TC samples between PTB and the HC. (c) STAMP results of lung samples between PTB and the HC. Fig. S4 Respiratory microbiota in HC: site-specific composition and distribution. (a) Common and specific species of Venn diagrams of in each part in the HC group. (b) Stack diagram showing the relative abundance of the top 20 species in each sample of PTB patients. (c) STAMP results of OS, TC, HS samples in HC individuals (statistical method: KW test). [file 12866_2025_4206_MOESM1_ESM.zip › Fig S1-4/Fig S4.tif]
