# Supplementary material for: Dysbiosis associated with enhanced microbial mobility across the respiratory tract in pulmonary tuberculosis patients
Source: BMC Microbiol. 2025 Aug 12;25:499. doi: 10.1186/s12866-025-04206-x (PMC12341359; doi:10.1186/s12866-025-04206-x)
Supplement: Supplementary file 2 — Supplementary Material 2. [file 12866_2025_4206_MOESM2_ESM.zip › Table S1-3/Table S2.doc]

**The top 20 species between current OS and published URT Microbiome (Shared Species Indicated by Shading).**

| **Current study top20 species** | **OS**  **abundance (%)** | **Other study top20 species** | **URT**  **abundance (%)** |
| --- | --- | --- | --- |
| *Haemophilus parainfluenzae* | 9.6767 | *Corynebacterium propinquum* | 9.5626 |
| *Prevotella melaninogenica* | 7.1835 | *Leptotrichia hongkongensis* | 6.8071 |
| *Rothia dentocariosa* | 5.4951 | *Pseudoalteromonas marina* | 5.9854 |
| *Rothia mucilaginosa* | 5.4013 | *Prevotella melaninogenica* | 4.6138 |
| *Neisseria subflava* | 4.7021 | *Acinetobacter variabilis* | 4.3087 |
| *Prevotella intermedia* | 4.1284 | *Bacteroides vulgatus* | 4.0927 |
| *Lautropia mirabilis* | 3.7530 | *Porphyromonas pasteri* | 3.3342 |
| *Schaalia odontolytica* | 2.3052 | *Ruminococcus bicirculans* | 3.2764 |
| *Rothia aeria* | 2.0343 | *Bacteroides stercoris* | 2.2675 |
| *Veillonella parvula* | 2.0318 | *Clostridium butyricum* | 2.1992 |
| *Neisseria sicca* | 1.8127 | *Corynebacterium imitans* | 2.0422 |
| *Prevotella nigrescens* | 1.7163 | *Bacteroides massiliensis* | 2.0230 |
| *Veillonella rogosae* | 1.5920 | *Bacteroides dorei* | 1.7247 |
| *Streptococcus oralis* | 1.5845 | *Acinetobacter indicus* | 1.7196 |
| *Prevotella jejuni* | 1.4868 | *Kocuria marina* | 1.6131 |
| *Streptococcus mitis* | 1.3867 | *Prevotella pallens* | 1.6117 |
| *Neisseria mucosa* | 1.3554 | *Kocuria palustris* | 1.6063 |
| *Actinomyces oris* | 1.2898 | *Streptococcus salivarius* | 1.4853 |
| *Actinomyces naeslundii* | 1.2221 | *Intestinimonas butyriciproducens* | 1.3694 |
| *Granulicatella adiacens* | 1.1784 | *Haemophilus haemolyticus* | 1.3013 |

**The top 20 species between current AS and published BALF Microbiome (Shared Species Indicated by Shading).**

| **Current study top20 species** | **AS**  **abundance (%)** | **Other study top20 species** | **BALF**  **abundance (%)** |
| --- | --- | --- | --- |
| *Prevotella melaninogenica* | 10.2261 | *Prevotella melaninogenica* | 12.5765 |
| *Prevotella jejuni* | 6.3671 | *Fusobacterium periodonticum* | 6.0010 |
| *Prevotella intermedia* | 5.8078 | *Leptotrichia hongkongensis* | 5.6744 |
| *Neisseria subflava* | 4.7723 | *Neisseria perflava* | 5.1016 |
| *Haemophilus parainfluenzae* | 4.0017 | *Haemophilus influenzae* | 3.5137 |
| *Prevotella nigrescens* | 3.6019 | *Neisseria elongata* | 3.2349 |
| *Veillonella rogosae* | 2.2186 | *Schaalia odontolytica* | 3.2168 |
| *Fusobacterium pseudoperiodonticum* | 2.1460 | *Prevotella pallens* | 3.1782 |
| *Rothia dentocariosa* | 1.8920 | *Corynebacterium propinquum* | 2.7284 |
| *Rothia mucilaginosa* | 1.6812 | *Capnocytophaga leadbetteri* | 2.1615 |
| *Schaalia odontolytica* | 1.6060 | *Campylobacter showae* | 2.0177 |
| *Campylobacter concisus* | 1.4131 | *Porphyromonas pasteri* | 1.9365 |
| *Veillonella atypica* | 1.2166 | *Campylobacter concisus* | 1.7410 |
| *Lautropia mirabilis* | 1.1425 | *Alloprevotella tannerae* | 1.6212 |
| *Prevotella histicola* | 1.1145 | *Prevotella heparinolytica* | 1.5998 |
| *Prevotella sp. oral taxon 299* | 1.0723 | *Prevotella jejuni* | 1.4443 |
| *Veillonella parvula* | 0.9174 | *Capnocytophaga sputigena* | 1.4420 |
| *Actinomyces oris* | 0.8740 | *Prevotella salivae* | 1.3930 |
| *Prevotella fusca* | 0.7754 | *Prevotella shahii* | 1.3903 |
| *Candidatus Nanosynbacter sp. HMT-352* | 0.6716 | *Prevotella denticola* | 1.3071 |
